# Independent occurrence of de novo *HSPD1* and *HIP1* variants in brothers with different neurological disorders – leukodystrophy and autism

**DOI:** 10.1038/s41439-018-0020-z

**Published:** 2018-07-19

**Authors:** Toshiyuki Yamamoto, Keiko Yamamoto-Shimojima, Yuki Ueda, Katsumi Imai, Yukitoshi Takahashi, Eri Imagawa, Noriko Miyake, Naomichi Matsumoto

**Affiliations:** 1 Institute of Medical Genetics, Tokyo Women’s Medical University, Tokyo, Japan; 2 Tokyo Women’s Medical University Institute for Integrated Medical Sciences, Tokyo, Japan; 3 Epilepsy Centre, NHO Shizuoka Institute of Epilepsy and Neurological Disorders, Shizuoka, Japan; 4 Department of Genetics, Yokohama City University, Yokohama, Japan

## Abstract

Consecutive occurrence of de novo variants in the same family is an extremely rare phenomenon. Two siblings, a younger brother with hypomyelinating leukodystrophy and an elder brother with severe intellectual disability and autistic features, had independent de novo variants of *HSPD1* c.139T > G (p.Leu47Val) and *HIP1* c.1393G > A (p.Glu465Lys), respectively. These novel variants were predicted to be pathogenic. Both patients also had a known *MECP2* variant, c.499C > T (p.Arg167Trp).

Hypomyelinating leukodystrophies (HLDs) are caused by congenital dysfunctions of the oligodendroglia and/or astrocytes^1^. Patients with HLDs show motor developmental delay and other neurological symptoms, including intellectual disability (ID), nystagmus, tremor, and epilepsy. Brain magnetic resonance imaging (MRI) demonstrates –T2-high intensity in the white matter^2^. OMIM (https://www.omim.org/) registers 13 subgroups of HLDs (Supplemental_Table_S1)^3,4^. Pelizaeus-Merzbacher disease (PMD; MIM#31208) is the most common HLD in male patients and is classified as HLD1. Because the proteolipid protein 1 gene (*PLP1*) responsible for PMD is located on the X-chromosome, PMD is an X-linked recessive disease.

Among HLDs, only HLD6 (MIM#612438) is related to autosomal dominant traits. Most patients with HLD6 have de novo variants in the tubulin beta-4A gene (*TUBB4A*)^5^, whereas *TUBB4A* is also related to autosomal dominant torsion dystonia 4 (DYT4; MIM#128101). In families with DYT4, *TUBB4A* variants are inherited as autosomal dominant traits. Thus, HLD6 and DYT4 are recognized as allelic disorders. The clinical difference between HLD6 and DYT4 is unclear, and there is an intermediate pattern^5^. Similarly, the 60-kDa heat shock protein 1 gene (*HSPD1*), responsible for HLD4 (MIM#612233)^6^, is associated with spastic paraplegia 13 (SPG13; MIM#605280)^7^, suggesting that HLD4 and SPG13 are also allelic disorders.

Here, we report on a de novo heterozygous *HSPD1* variant in a sporadic patient with HLD4 but not SPG13 (patient 1, Supplemental Information) in association with a de novo variant in the huntingtin-interacting protein 1 gene (*HIP1*), which was concurrently identified in his elder brother (patient 2, Supplemental Information).

In accordance with the declaration of Helsinki, this study was approved by the ethics committee of Tokyo Women’s Medical University. After receiving written informed consent from the family, we obtained blood samples from siblings and their parents. Genomic DNA was extracted for sequence analysis and used for genetic diagnosis.

Because PMD derived from duplications or single-nucleotide alterations in the *PLP1* region is the major type of HLD, *PLP1* analysis by microarray-based comparative genomic hybridization and Sanger sequencing was performed as the first tier^8^, and these confirmed no abnormalities. Whole-exome sequencing (WES) was performed for both parents and siblings (patients 1 and 2) as described^9^. Synonymous variants, variants with more than 1% global population frequency, and variants registered in the Human Genetic Variation Database^10^ were filtered. Variants with de novo origin or those showing Mendelian inheritance patterns, associated with autosomal dominant or recessive traits, were manually selected. Pathogenic genomic copy-number variations (CNVs) were not identified by the eXome Hidden Markov Model using the data extracted through WES^11^.

Novel variants, NM_002156.4(HSPD1):c.139T > G (p.Leu47Val) and NM_005338.6(HIP1):c.1393G > A (p.Glu465Lys), were identified in patients 1 and 2, respectively. These variants were not registered in the 1KGP, ExAC (http://exac.broadinstitute.org/), ESP6500 (http://evs.gs.washington.edu/EVS/), or iJGVD database (Supplemental Table S3, 4)^12,13^. At the same position on *HIP1* c.1393G > A, there is a known SNP, rs782598703G > C (p.Glu465Gln) (location chr7:75187292), with a minor allele frequency of 0.001% (1/121394). Both patients and their mother carried NM_004992.3(MECP2):c.499C > T (p.Arg167Trp), registered as rs61748420 (Supplemental Table S2, 5), indicating X-linked recessive inheritance. There was no other possible candidate variant in the genes related to the clinical features in association with Mendelian inheritance, including autosomal-recessive traits. The results are summarized in Table 1, together with clinical features. All variants were confirmed by Sanger sequencing (Fig._1c), and damaging scores predicted through wANNOVAR (http://wannovar.wglab.org/) are listed in Supplemental_Table_S2. Most of the scores, including CADD, suggested pathogenicity. The results of wANNOVAR analysis for all previously reported *HSPD1* variants are compared in Supplemental_Table_S3, suggesting no significant difference from the present variant. The amino acid sequences affected by all variants discussed in this study are conserved among species (Supplemental_Figure_S1).

**Table 1:** Summary of the clinical features and gene variants

		Patient 1	Patient 2
Gender		Male	Male
Age		8 years	12 years
Stature at birth			
	Weight (g)	2368	2470
	OFC (cm)	29	33
Stature at last examination			
	Height	[−4.0SD]	[−2.8SD]
	Weight	[−2.3SD]	[−2.1SD]
Early motor developmental delay		Mild	Moderate
	Standing/walking	12 mo.	20 mo.
Febrile seizures		+	+
Intractable seizures		+	−
Pyramidal sign		+	−
Dysarthria		+	−
Cerebellar signs		+	−
Developmental deterioration		+	−
Abnormal MRI findings		+	-
Cognitive impairment		−	+
Intellectual disability		−	+
Severe autistic features		−	+
Gene variants			
	*HSPD1* p.Leu47Val	+	−
	*HIP1* p.Glu465Lys	−	+
	*MECP2* p.Arg167Trp	+	+

*OFC* occipito-frontal circumference, *SD* standard deviation, *mo* months

**Fig. 1:**
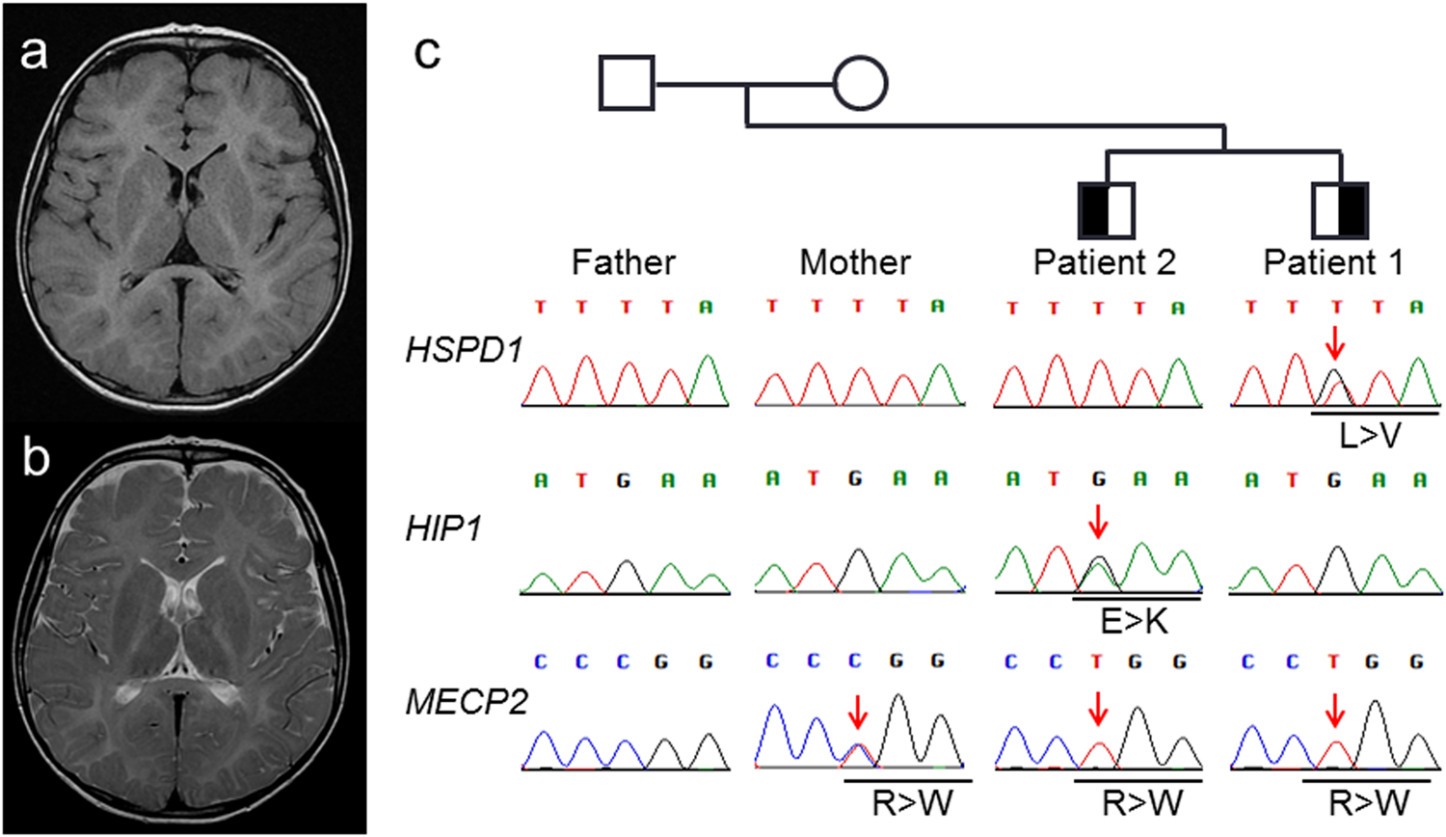
Results of brain MRI and molecular investigations. T1- (**a**) and T2-weighted axial images (**b**) of patient 1 examined at 4 years old. A –T2-high-signal is noted in the white matter, indicating hypomyelination. **c** Electropherograms of Sanger sequencing depicted in the family tree. Variants in *HSPD1* and *HIP1* are identified only in patient 1 and patient 2, respectively. Alternatively, the *MECP2* variant is identified in both patients as hemizygous and in their mother as heterozygous, indicating an X-linked recessive variant

Protein secondary structures were predicted through web-based software provided by PRABI-Lyon-Gerland (The Institute of Biology and Chemistry of Proteins, Lyon, France; https://prabi.ibcp.fr/htm/site/web/home). A marked change was shown by *HSPD1* p.Leu47Val but not by *HIP1* p.Glu465Lys (Supplemental Figure S2,3).

Patient 1 showed progressive paraplegia due to unknown leukodystrophy (Fig._1a, b), and HLD4 was considered as a clinical diagnosis rather than SPG13. Although bi-allelic *HSPD1* involvement was expected^6^, only a hemi-allelic involvement was confirmed. This may have reflected the existence of an unidentified variant in the non-coding region of *HSPD1*, because pathogenic CNVs were not observed. Another possible explanation is that HLD4 in this patient was caused by hemi-allelic involvement of *HSPD1*.

An example of a gene related to both autosomal-recessive and dominant traits is the hepatic and glial cell adhesion molecule gene (*HEPACAM*)^14^. Megalencephalic leukoencephalopathy with subcortical cysts 2A and B (MLC2A MIM#613925; MLC2B MIM#613926) is related to *HEPACAM* variants in recessive and dominant patterns, respectively. Although HLD4 and SPG13 are considered to be inherited through different modes, with autosomal-recessive and dominant traits, the main clinical features are commonly derived from pyramidal tract involvement owing to the dysfunction of myelin. Therefore, HLD4 and SPG13 may be involved in the same clinical spectrum, and the HLD4 in this patient may have been derived from hemi-allelic involvement in *HSPD1*, as in SPG13.

Only four *HSPD1* variants, pVal98Ile^7^, p.Gln461Glu^15^, and p.Gly563Ala^16^, have been reported as pathogenic variants for the autosomal dominant trait of SPG13, and only one homozygous variant, p.Asp29Gly, has been reported for the autosomal-recessive trait of HLD4^6^, indicating that bi-allelic involvement is rare. In our in silico analysis of the novel variant of p.Leu47Val, a marked conformational change of HSPD1 protein was predicted. Thus, a dominant negative effect of p.Leu47Val was suspected, and it may have been responsible for the autosomal dominant trait of HLD4.

In contrast to patient 1, patient 2 did not show any abnormal findings on brain MRI. This is reasonable because patient 2 did not have the *HSPD1* variant. Rather, he showed severe neuropsychiatric features, including ID, severe autistic features, and intractable epilepsy. WES identified a de novo *HIP1* (the huntingtin-interacting protein 1 gene) variant. *HIP1* is located on the neighboring region of the William syndrome critical region in 7q11.23, and atypically large deletions in 7q11.23 including *HIP1* have been identified in several patients^17–19^. Based on genotype-phenotype correlation studies for such cases, *HIP1* has been considered a candidate gene for neurological impairments. This is also supported by several functional studies^20,21^. The de novo heterozygous *HIP1* variant identified in this study may suggest the previously considered hypothesis that *HIP1* is responsible for neurological impairments, although no marked protein conformational change was identified with a prediction tool.

In the present sibling cases, a previously reported *MECP2* (the methyl CpG binding protein 2 gene) variant, p.Arg167Trp, was commonly observed as an X-linked recessive trait. This variant was previously identified in a three-generation family consisting of four individuals with non-specific X-linked ID^22^. Thus, p.Arg167Trp may have some clinical impact on the present sibling cases; however, patient 1 did not show developmental delay in the early stage. Thus, the severe neurological manifestations observed in patient 2 cannot be explained by the *MECP2* p.Arg167Trp variant.

De novo variants are the major cause of neurodevelopmental disorders in childhood. In such cases, probands in their families are recognized as sporadic cases. Although de novo variants can be observed with an estimated background rate of 0.86 amino-acid-altering variants per newborn in controls^23^, it would be rare for de novo variants to have a pathogenic impact on the proteins related to important functions. Thus, consecutive occurrence of de novo variants in the same family is an extremely rare phenomenon. Only a few cases of such consecutive occurrence of de novo variants have been reported^24^. Here, we observed different de novo variants in sibling cases with different phenotypic features.

## Electronic supplementary material

Supplemental Information

## Data Availability

The relevant data from this Data Report are hosted at the Human Genome Variation Database at: 10.6084/m9.figshare.hgv.2348; 10.6084/m9.figshare.hgv.2351; 10.6084/m9.figshare.hgv.2354
