# Supplementary material for: Independent occurrence of de novo HSPD1 and HIP1 variants in brothers with different neurological disorders – leukodystrophy and autism
Source: Hum Genome Var. 2018 Jul 19;5:18. doi: 10.1038/s41439-018-0020-z (PMC6053359; doi:10.1038/s41439-018-0020-z)
Supplement: Supplementary file 1 — Supplemental Information [file 41439_2018_20_MOESM1_ESM.pdf]

Supplemental information

"Independent occurrence of *de novo* variants in *HSPD1* and *HIP1*"

Independent occurrence of *de novo HSPD1* and *HIP1* variants identified in male brothers with different neurological disorders – leukodystrophy and autism

Toshiyuki Yamamoto[1,2)], Keiko Yamamoto-Shimojima[1,2)], Yuki Ueda[3)], Katsumi Imai[3)], Yukitoshi Takahashi[3)], Eri Imagawa[4)], Noriko Miyake[4)], Naomichi Matsumoto[4)]

1) Institute of Medical Genetics, Tokyo Women's Medical University, Tokyo, Japan

2) Tokyo Women's Medical University Institute for Integrated Medical Sciences, Tokyo, Japan

3) Epilepsy Centre, NHO Shizuoka Institute of Epilepsy and Neurological Disorders, Shizuoka, Japan

4) Department of Genetics, Yokohama City University, Yokohama, Japan

Patients report

A male patient (patient 1) was born at term with a birth weight of 2368 g (3-10th percentile) and an occipito-frontal circumference (OFC) of 29 cm (<3rd percentile). In early infancy, this patient showed failure-to-thrive and mild motor developmental delay with head control at 5 months. Febrile seizures were observed since 6 months. He

started to stand with support at 12 month; however, nystagmus and intension tremor were noted since this time. Then, developmental deterioration was gradually manifested. His maximum developmental level was that observed at 12 months. Brain magnetic resonance imaging (MRI) examined at 4 years showed T2-high intensity in the deep white matter (Fig. 1A, B). Auditory brainstem response showed normal patterns. Electroencephalogram (EEG) showed bi-frontal continuous spikes. Routine laboratory examination including thyroid function showed no abnormality. Chromosomal G-banding showed a normal male karyotype of 46,XY. Metabolic screening showed no abnormality.

At 5 years, he lost his ability to sit alone owing to remarkable nystagmus and intention tremor. He understood the concept of color and number. Although there was dysarthria, he could speak 2 word sentences. At 7 years, his height was 102 cm (<3rd percentile) and weight was 14.4 kg (<3rd percentile), indicating severe growth failure. There were hyperreflexia and abnormal reflexes including clonus and Babinski, indicating spastic paraplegia. Intractable seizures were daily observed and dysphagia became apparent. He required supports for dairy life. After participated in this study, this patient died at his age of 8 years due to heart failure.

His elder brother (patient 2) is 12 years old. He also shows neurodevelopmental

disorder since early infancy. Patient 2 was born at term with a birth weight of 2470 g (3-10th percentile), length of 47 cm (10-25th percentile), and OFC of 33 cm (25-50th percentile). He started to show febrile seizures since 6 months. He showed gross motor development delay with sitting since 8 months and walking since 20 months. In comparison with his moderate motor developmental delay, his language development was remarkably delayed. Since his age of 5 years, he showed focal seizures with focal spikes in the central region revealed by EEG.

At 12 years, his height and weight were 128.4 cm (<3rd percentile) and 26.2 kg (<3rd percentile), respectively, indicating growth failure. In comparison with patient 1, there was no abnormality in the motor function. This elder brother showed no abnormality in brain MRI. Although he can speak some words, his communication skill is extremely poor and he requires help for his whole dairy activities, indicating severe intellectual disability. Because he often shows hyperkinetic behavior, panic, and insomnia, he was diagnosed as having severe autistic features.

Supplemental information

"Independent occurrence of *de novo* variants in *HSPD1* and *HIP1*"

Supplemental Table S1. A list of the HLDs registered in OMIM

	disease MIM	genes	inheritance trait	gene MIM
HLD1	#312080	*PLP1*	XLR	#300401
HLD2	#608804	*GJC2*	AR	#608803
HLD3	#260600	*AIMP1*	AR	#603605
HLD4	#612233	*HSPD1*	AR	#118190
HLD5	#610532	*FAM126A*	AR	#610531
HLD6	#612438	*TUBB4A*	AD	#602662
HLD7	#607694	*POLR3A*	AR	#614258
HLD8	#614381	*POLR3B*	AR	#614366
HLD9	#616140	*RARS*	AR	#107820
HLD10	#616420	*PYCR2*	AR	#616406
HLD11	#616494	*POLR1C*	AR	#610060
HLD12	#616683	*VPS11*	AR	#608549
HLD13	#616881	*HIKESHI*	AR	#614908

Supplemental information

"Independent occurrence of *de novo* variants in *HSPD1* and *HIP1*"

Supplemental Table S2. Summary of the prediction scores			
	Patient 1	Patient 2	Patient 1 & 2
	HSPD1	*HIP1*	*MECP2*
Nucleotide changes	NM_002156.4:c.139T>G	NM_005338.6:c.1393G>A	NM_004992.3:c.499C>T
Amino-acid changes	NM_002156.4:p.(Leu47Val)	NM_005338.6:p.(Glu465Lys)	NM_004992.3:p.(Arg167Trp)
dbSNP	Not registered	Not registered	rs61748420
SIFT_score	0.001	0.002	0.000
Polyphen2_HDIV_score	0.974	0.989	0.899
CADD_phred	24.3	28.0	31.0
ToMMo iJGVD database	Not registered	Not registered	NA*
*, Variants data in chromosome X are not included.			

Supplemental information

"Independent occurrence of *de novo* variants in *HSPD1* and *HIP1*"

Supplemental Table S3. Results of in-silico analysis for *HSPD1* mutations through wANNOVAR					
	Magen et al., 2008	Present case	Hansen et al., 2002	Hansen et al., 2007	Hewamadduma et al., 2008
	c.86A>G	c.139T>G	c.292G>A	c.1381C>G	c.1688G>C
	p.D29G	p.L47V	p.V98I	p.Q461E	p.G563A
Chr	2	2	2	2	2
Start	198363487	198363434	198361999	198353050	198351804
End	198363487	198363434	198361999	198353050	198351804
Ref	T	A	C	G	C
Alt	C	C	T	C	G
Func.refGene	exonic	exonic	exonic	exonic	exonic
Gene.refGene	HSPD1	HSPD1	HSPD1	HSPD1	HSPD1
GeneDetail.refGene					
ExonicFunc.refGene	nonsynonymous SNV	nonsynonymous SNV	nonsynonymous SNV	nonsynonymous SNV	nonsynonymous SNV
AAChange.refGene(HSPD1:NM_002156)	exon2:c.A86G:p.D29G	exon2:c.T139G:p.L47V	exon3:c.G292A:p.V98I	exon10:c.C1381G:p.Q461E	exon12:c.G1688C:p.G563A
AAChange.refGene(HSPD1:NM_199440)	exon2:c.A86G:p.D29G	exon2:c.T139G:p.L47V	exon3:c.G292A:p.V98I	exon10:c.C1381G:p.Q461E	exon12:c.G1688C:p.G563A
1000G_ALL	.	.	.	.	0.0076
1000G_AFR	.	.	.	.	0.0008
1000G_AMR	.	.	.	.	0.0086
1000G_EAS	.	.	.	.	.
1000G_EUR	.	.	.	.	0.026
1000G_SAS	.	.	.	.	0.0051
ExAC_Freq	.	.	.	.	0.0168
ExAC_AFR	.	.	.	.	0.0022
ExAC_AMR	.	.	.	.	0.0083
ExAC_EAS	.	.	.	.	0.0004
ExAC_FIN	.	.	.	.	0.0496
ExAC_NFE	.	.	.	.	0.0205
ExAC_OTH	.	.	.	.	0.0228
ExAC_SAS	.	.	.	.	0.0109
ESP6500si_ALL	.	.	.	.	0.012
ESP6500si_AA	.	.	.	.	0.0031
ESP6500si_EA	.	.	.	.	0.017
CG46	.	.	.	.	0.011
NCI60	.	.	.	.	0.0082
dbSNP	rs72466451	.	rs66468541	.	rs41265953
COSMIC_ID	.	.	.	.	COSM6049720
COSMIC_DIS	.	.	.	.	1(large_intestine)
ClinVar_SIG	Pathogenic	.	Pathogenic	.	Benign|Benign|Likely benign
ClinVar_DIS	Leukodystrophy¥x2c_hypomyelinating¥x2c_4	.	Spastic_paraplegia_13	.	not_specified|Spastic_paraplegia|Spastic_Paraplegia¥x2c_Dominant
ClinVar_ID	RCV000019113.25	.	RCV000019112.28	.	RCV000125388.1|RCV000233769.2|RCV000369197.1
ClinVar_DB	MedGen:OMIM:Orphanet	.	MedGen:OMIM:Orphanet	.	MedGen|MedGen|MedGen
ClinVar_DBID	C2677109:612233:ORPHA280288	.	C1854467:605280:ORPHA100994	.	CN169374|C0037772|CN239430
GWAS_DIS	.	.	.	.	.
GWAS_OR	.	.	.	.	.
GWAS_BETA	.	.	.	.	.
GWAS_PUBMED	.	.	.	.	.
GWAS_SNP	.	.	.	.	.
GWAS_P	.	.	.	.	.
SIFT_score	0.051	0.001	0.079	0.064	0
SIFT_converted_rankscore	0.44	0.912	0.446	0.365	0.912
SIFT_pred	T	D	T	T	D
Polyphen2_HDIV_score	0.008	0.974	0.248	0.036	0.999
Polyphen2_HDIV_rankscore	0.17	0.715	0.368	0.615	0.764
Polyphen2_HDIV_pred	B	D	B	B	D
Polyphen2_HVAR_score	0.01	0.688	0.393	0.14	0.981
Polyphen2_HVAR_rankscore	0.341	0.807	0.49	0.807	0.736
Polyphen2_HVAR_pred	B	P	B	B	D
LRT_score	0	0	0	0	0
LRT_converted_rankscore	0.843	0.843	0.629	0.843	0.843
LRT_pred	D	D	D	D	D
MutationTaster_score	1	1	1	1	1
MutationTaster_converted_rankscore	0.81	0.588	0.81	0.81	0.81
MutationTaster_pred	A	D	A	D	D
MutationAssessor_score	3.195	2.85	2.575	1.05	2.87
MutationAssessor_score_rankscore	0.893	0.831	0.755	0.269	0.836
MutationAssessor_pred	M	M	M	L	M
FATHMM_score	-0.47	-1.69	-1.11	-0.97	-0.75
FATHMM_converted_rankscore	0.776	0.83	0.775	0.757	0.733
FATHMM_pred	T	D	T	T	T
PROVEAN_score	-4.42	-2.51	-0.84	-1.73	-4.14
PROVEAN_converted_rankscore	0.887	0.608	0.236	0.41	0.752
PROVEAN_pred	D	D	N	N	D
VEST3_score	0.277	0.37	0.133	0.848	0.245
VEST3_rankscore	0.965	0.831	0.79	0.835	0.299
MetaSVM_score	0.114	0.28	0.268	-0.046	-0.166
MetaSVM_rankscore	0.844	0.872	0.87	0.813	0.785
MetaSVM_pred	D	D	D	T	T
MetaLR_score	0.505	0.671	0.557	0.452	0.295
MetaLR_rankscore	0.813	0.886	0.839	0.786	0.666
MetaLR_pred	D	D	D	T	T
M-CAP_score	0.139	0.129	0.047	0.077	.
M-CAP_rankscore	0.822	0.812	0.628	0.729	.
M-CAP_pred	D	D	D	D	.
CADD_raw	4.44	4.519	3.725	3.275	4.167
CADD_raw_rankscore	0.594	0.605	0.505	0.452	0.559
CADD_phred	24.2	24.3	23.3	22.8	23.8
DANN_score	0.996	0.998	0.998	0.988	0.99
DANN_rankscore	0.741	0.849	0.897	0.464	0.495
fathmm-MKL_coding_score	0.86	0.578	0.978	0.979	0.95
fathmm-MKL_coding_rankscore	0.449	0.302	0.773	0.782	0.627
fathmm-MKL_coding_pred	D	D	D	D	D

Continue

	Magen et al., 2008	Present case	Hansen et al., 2002	Hansen et al., 2007	Hewamadduma et al., 2008
	c.86A>G	c.139T>G	c.292G>A	c.1381C>G	c.1688G>C
	p.D29G	p.L47V	p.V98I	p.Q461E	p.G563A
Eigen_coding_or_noncoding	c	c	c	c	c
Eigen-raw	0.241	0.379	0.389	0.358	0.725
Eigen-PC-raw	0.348	0.274	0.441	0.506	0.717
GenoCanyon_score	1	1	1	1	1
GenoCanyon_score_rankscore	0.747	0.747	0.747	0.747	0.747
integrated_fitCons_score	0.733	0.733	0.628	0.672	0.707
integrated_fitCons_score_rankscore	0.969	0.969	0.401	0.522	0.73
integrated_confidence_value	0	0	0	0	0
GERP++_RS	4.87	1.22	4.24	5.39	5.22
GERP++_RS_rankscore	0.627	0.202	0.493	0.775	0.722
phyloP100way_vertebrate	7.986	2.24	7.879	9.4	6.979
phyloP100way_vertebrate_rankscore	0.877	0.425	0.856	0.966	0.758
phyloP20way_mammalian	1.011	0.189	0.852	0.98	0.935
phyloP20way_mammalian_rankscore	0.635	0.248	0.362	0.597	0.49
phastCons100way_vertebrate	1	0.995	1	1	1
phastCons100way_vertebrate_rankscore	0.715	0.385	0.715	0.715	0.715
phastCons20way_mammalian	1	0.996	1	0.998	0.983
phastCons20way_mammalian_rankscore	0.888	0.625	0.888	0.697	0.502
SiPhy_29way_logOdds	14.756	9.066	17.246	19.504	19.2
SiPhy_29way_logOdds_rankscore	0.69	0.354	0.869	0.951	0.937
Interpro_domain	GroEL-like equatorial domain	GroEL-like equatorial domain	GroEL-like equatorial domain	GroEL-like equatorial domain	.
GTEx_V6_gene	.	.	.	.	.
GTEx_V6_tissue	.	.	.	.	.
gnomAD_exome_ALL	.	.	.	.	0.017
gnomAD_exome_AFR	.	.	.	.	0.0022
gnomAD_exome_AMR	.	.	.	.	0.0067
gnomAD_exome_ASJ	.	.	.	.	0.0147
gnomAD_exome_EAS	.	.	.	.	0.0002
gnomAD_exome_FIN	.	.	.	.	0.0498
gnomAD_exome_NFE	.	.	.	.	0.0201
gnomAD_exome_OTH	.	.	.	.	0.0155
gnomAD_exome_SAS	.	.	.	.	0.0109
gnomAD_genome_ALL	.	.	.	.	0.0164
gnomAD_genome_AFR	.	.	.	.	0.0029
gnomAD_genome_AMR	.	.	.	.	0.006
gnomAD_genome_ASJ	.	.	.	.	0.0232
gnomAD_genome_EAS	.	.	.	.	0
gnomAD_genome_FIN	.	.	.	.	0.0489
gnomAD_genome_NFE	.	.	.	.	0.0182
gnomAD_genome_OTH	.	.	.	.	0.0286
Otherinfo					

Supplemental information

"Independent occurrence of *de novo* variants in *HSPD1* and *HIP1*"

Supplemental Table S4. Results of in-silico analysis for *HIP1* mutation through wANNOVAR

	Patient 2
	c.1393A>G
	p.E465K
Chr	7
Start	75187542
End	75187542
Ref	C
Alt	T
Func.refGene	exonic
Gene.refGene	HIP1
GeneDetail.refGene	
ExonicFunc.refGene	nonsynonymous SNV
AAChange.refGene	HIP1:NM_001243198:exon15:c.G1393A:p.E465K, HIP1:NM_005338:exon15:c.G1393A:p.E465K
1000G_ALL	.
1000G_AFR	.
1000G_AMR	.
1000G_EAS	.
1000G_EUR	.
1000G_SAS	.
ExAC_Freq	.
ExAC_AFR	.
ExAC_AMR	.
ExAC_EAS	.
ExAC_FIN	.
ExAC_NFE	.
ExAC_OTH	.
ExAC_SAS	.
ESP6500si_ALL	.
ESP6500si_AA	.
ESP6500si_EA	.
CG46	.
NCI60	.
dbSNP	.
COSMIC_ID	.
COSMIC_DIS	.
ClinVar_SIG	.
ClinVar_DIS	.
ClinVar_ID	.
ClinVar_DB	.
ClinVar_DBID	.
GWAS_DIS	.
GWAS_OR	.
GWAS_BETA	.
GWAS_PUBMED	.
GWAS_SNP	.
GWAS_P	.
SIFT_score	0.002
SIFT_converted_rankscore	0.721
SIFT_pred	D
Polyphen2_HDIV_score	0.989
Polyphen2_HDIV_rankscore	0.609
Polyphen2_HDIV_pred	D
Polyphen2_HVAR_score	0.703
Polyphen2_HVAR_rankscore	0.524
Polyphen2_HVAR_pred	P
LRT_score	0
LRT_converted_rankscore	0.843
LRT_pred	D
MutationTaster_score	1
MutationTaster_converted_rankscore	0.81
MutationTaster_pred	D
MutationAssessor_score	3.225
MutationAssessor_score_rankscore	0.897
MutationAssessor_pred	M
FATHMM_score	2.59
FATHMM_converted_rankscore	0.162
FATHMM_pred	T
PROVEAN_score	-3.57
PROVEAN_converted_rankscore	0.69
PROVEAN_pred	D
VEST3_score	0.831
VEST3_rankscore	0.822
MetaSVM_score	-0.997
MetaSVM_rankscore	0.308
MetaSVM_pred	T
MetaLR_score	0.101
MetaLR_rankscore	0.374
MetaLR_pred	T
M-CAP_score	0.051
M-CAP_rankscore	0.646
M-CAP_pred	D
CADD_raw	6.047
CADD_raw_rankscore	0.832
CADD_phred	28
DANN_score	0.999
DANN_rankscore	0.968
fathmm-MKL_coding_score	0.988
fathmm-MKL_coding_rankscore	0.863
fathmm-MKL_coding_pred	D

Continue

	Patient 2<br>c.1393A>G<br>p.E465K
Eigen_coding_or_noncoding	c
Eigen-raw	0.721
Eigen-PC-raw	0.709
GenoCanyon_score	1
GenoCanyon_score_rankscore	0.747
integrated_fitCons_score	0.707
integrated_fitCons_score_rankscore	0.73
integrated_confidence_value	0
GERP++_RS	5.18
GERP++_RS_rankscore	0.71
phyloP100way_vertebrate	7.876
phyloP100way_vertebrate_rankscore	0.855
phyloP20way_mammalian	0.805
phyloP20way_mammalian_rankscore	0.326
phastCons100way_vertebrate	1
phastCons100way_vertebrate_rankscore	0.715
phastCons20way_mammalian	0.957
phastCons20way_mammalian_rankscore	0.436
SiPhy_29way_logOdds	17.695
SiPhy_29way_logOdds_rankscore	0.881
Interpro_domain	.
GTEx_V6_gene	.
GTEx_V6_tissue	.
gnomAD_exome_ALL	.
gnomAD_exome_AFR	.
gnomAD_exome_AMR	.
gnomAD_exome_ASJ	.
gnomAD_exome_EAS	.
gnomAD_exome_FIN	.
gnomAD_exome_NFE	.
gnomAD_exome_OTH	.
gnomAD_exome_SAS	.
gnomAD_genome_ALL	.
gnomAD_genome_AFR	.
gnomAD_genome_AMR	.
gnomAD_genome_ASJ	.
gnomAD_genome_EAS	.
gnomAD_genome_FIN	.
gnomAD_genome_NFE	.
gnomAD_genome_OTH	.
Otherinfo	

Supplemental information

"Independent occurrence of *de novo* variants in *HSPD1* and *HIP1*"

Supplemental Table S5. Results of in-silico analysis for *MECP2* mutation through wANNOVAR	
	Patient 1 & 2
	c.499C>T
	p.R167W
Chr	X
Start	153296780
End	153296780
Ref	G
Alt	A
Func.refGene	exonic
Gene.refGene	MECP2
GeneDetail.refGene	
ExonicFunc.refGene	nonsynonymous SNV
AAChange.refGene	MECP2:NM_001110792:exon3:c.C535T:p.R179W,<br>MECP2:NM_004992:exon4:c.C499T:p.R167W,<br>MECP2:NM_001316337:exon5:c.C220T:p.R74W
1000G_ALL	.
1000G_AFR	.
1000G_AMR	.
1000G_EAS	.
1000G_EUR	.
1000G_SAS	.
ExAC_Freq	.
ExAC_AFR	.
ExAC_AMR	.
ExAC_EAS	.
ExAC_FIN	.
ExAC_NFE	.
ExAC_OTH	.
ExAC_SAS	.
ESP6500si_ALL	.
ESP6500si_AA	.
ESP6500si_EA	.
CG46	.
NCI60	.
dbSNP	rs61748420
COSMIC_ID	.
COSMIC_DIS	.
ClinVar_SIG	Uncertain significance|Pathogenic
ClinVar_DIS	Mental_retardation¥x2c_X-linked¥x2c_syndromic_13|Rett_syndrome
ClinVar_ID	RCV000133142.2|RCV000193537.1
ClinVar_DB	MedGen:OMIM:Orphanet|MedGen:OMIM:Orphanet:SNOMED_CT
ClinVar_DBID	C1968550:300055:ORPHA3077|C0035372:312750:ORPHA778:68618008
GWAS_DIS	.
GWAS_OR	.
GWAS_BETA	.
GWAS_PUBMED	.
GWAS_SNP	.
GWAS_P	.
SIFT_score	0
SIFT_converted_rankscore	0.912
SIFT_pred	D
Polyphen2_HDIV_score	1
Polyphen2_HDIV_rankscore	0.899
Polyphen2_HDIV_pred	D
Polyphen2_HVAR_score	0.997
Polyphen2_HVAR_rankscore	0.85
Polyphen2_HVAR_pred	D
LRT_score	0
LRT_converted_rankscore	0.843
LRT_pred	D
MutationTaster_score	1
MutationTaster_converted_rankscore	0.81
MutationTaster_pred	D
MutationAssessor_score	2.25
MutationAssessor_score_rankscore	0.64
MutationAssessor_pred	M
FATHMM_score	-4.03
FATHMM_converted_rankscore	0.964
FATHMM_pred	D
PROVEAN_score	-3.75
PROVEAN_converted_rankscore	0.711
PROVEAN_pred	D
VEST3_score	0.891
VEST3_rankscore	0.905
MetaSVM_score	0.949
MetaSVM_rankscore	0.964
MetaSVM_pred	D
MetaLR_score	0.885
MetaLR_rankscore	0.962
MetaLR_pred	D
M-CAP_score	0.937
M-CAP_rankscore	0.996
M-CAP_pred	D
CADD_raw	6.523
CADD_raw_rankscore	0.899
CADD_phred	31
DANN_score	0.999
DANN_rankscore	0.976
fathmm-MKL_coding_score	0.98
fathmm-MKL_coding_rankscore	0.785
fathmm-MKL_coding_pred	D

Continue

	Patient 1 & 2
	c.499C>T
	p.R167W
Eigen_coding_or_noncoding	.
Eigen-raw	.
Eigen-PC-raw	.
GenoCanyon_score	1
GenoCanyon_score_rankscore	0.747
integrated_fitCons_score	.
integrated_fitCons_score_rankscore	.
integrated_confidence_value	.
GERP++_RS	4.59
GERP++_RS_rankscore	0.561
phyloP100way_vertebrate	7.476
phyloP100way_vertebrate_rankscore	0.799
phyloP20way_mammalian	0.006
phyloP20way_mammalian_rankscore	0.137
phastCons100way_vertebrate	1
phastCons100way_vertebrate_rankscore	0.715
phastCons20way_mammalian	0.999
phastCons20way_mammalian_rankscore	0.75
SiPhy_29way_logOdds	13.286
SiPhy_29way_logOdds_rankscore	0.595
Interpro_domain	DNA-binding domain|Methyl-CpG DNA binding
GTEx_V6_gene	.
GTEx_V6_tissue	.
gnomAD_exome_ALL	.
gnomAD_exome_AFR	.
gnomAD_exome_AMR	.
gnomAD_exome_ASJ	.
gnomAD_exome_EAS	.
gnomAD_exome_FIN	.
gnomAD_exome_NFE	.
gnomAD_exome_OTH	.
gnomAD_exome_SAS	.
gnomAD_genome_ALL	.
gnomAD_genome_AFR	.
gnomAD_genome_AMR	.
gnomAD_genome_ASJ	.
gnomAD_genome_EAS	.
gnomAD_genome_FIN	.
gnomAD_genome_NFE	.
gnomAD_genome_OTH	.
Otherinfo	

Supplemental information

"Independent occurrence of *de novo* variants in *HSPD1* and *HIP1*"

Supplemental Fig. S1. Conservation of the amino-acid sequence compared with other species

		Magen et al., 2008			Present case			Hansen et al., 2002			Hansen et al., 2007			Hewamadduma et al., 2008		
		c.86A>G			c.139T>G			c.292G>A			c.1381C>G			c.1688G>C		
		p.D29G			p.L47V			p.V98I			p.Q461E			p.G563A		
			⇓			⇓			⇓			⇓			⇓	
HSPD1	Human	K	D	V	L	L	A	L	V	Q	D	Q	K	G	G	M
	Rhesus	K	D	V	L	L	A	L	V	Q	D	Q	K	S	G	M
	Mouse	K	D	V	L	L	A	L	V	Q	D	Q	K	G	G	M
	Dog	K	D	V	L	L	A	L	V	Q	D	Q	R	G	G	M
	Elephant	K	D	V	L	L	A	L	V	Q	D	Q	K	G	G	M
	Chicken	K	D	V	L	L	A	L	V	Q	D	Q	K	G	G	M
	X_tropicalis	K	D	V	L	L	A	L	V	Q	D	Q	R	G	G	M
	Zebrafish	K	D	V	L	L	A	L	V	Q	D	Q	K	G	G	M
	Lamprey	K	D	V	L	L	A	L	V	Q	=	=	=	=	=	=

		Present case		
		c.1393G>A		
		p.E465K		
			⇓	
HIP1	Human	N	E	Q
	Rhesus	N	E	Q
	Mouse	N	E	Q
	Dog	N	E	Q
	Elephant	N	E	Q
	Chicken	N	E	Q
	X_tropicalis	N	E	Q
	Zebrafish	N	E	Q
	Lamprey	N	E	Q

Amino-acids affected by *HSPD1* mutations reported previously and reported in this study are compared with other species. The amino-acid affected by the *HIP1* mutation identified in this study is also compared with other species. The amino-acids which are different from other species are high-lighted by gray collumn. All amino-acids are conserved among species as shown.

Supplemental information
"Independent occurrence of *de novo* variants in *HSPD1* and *HIP1*"

Supplemental Figure S2. Results of the prediction for secondary structure of *HSPD1*

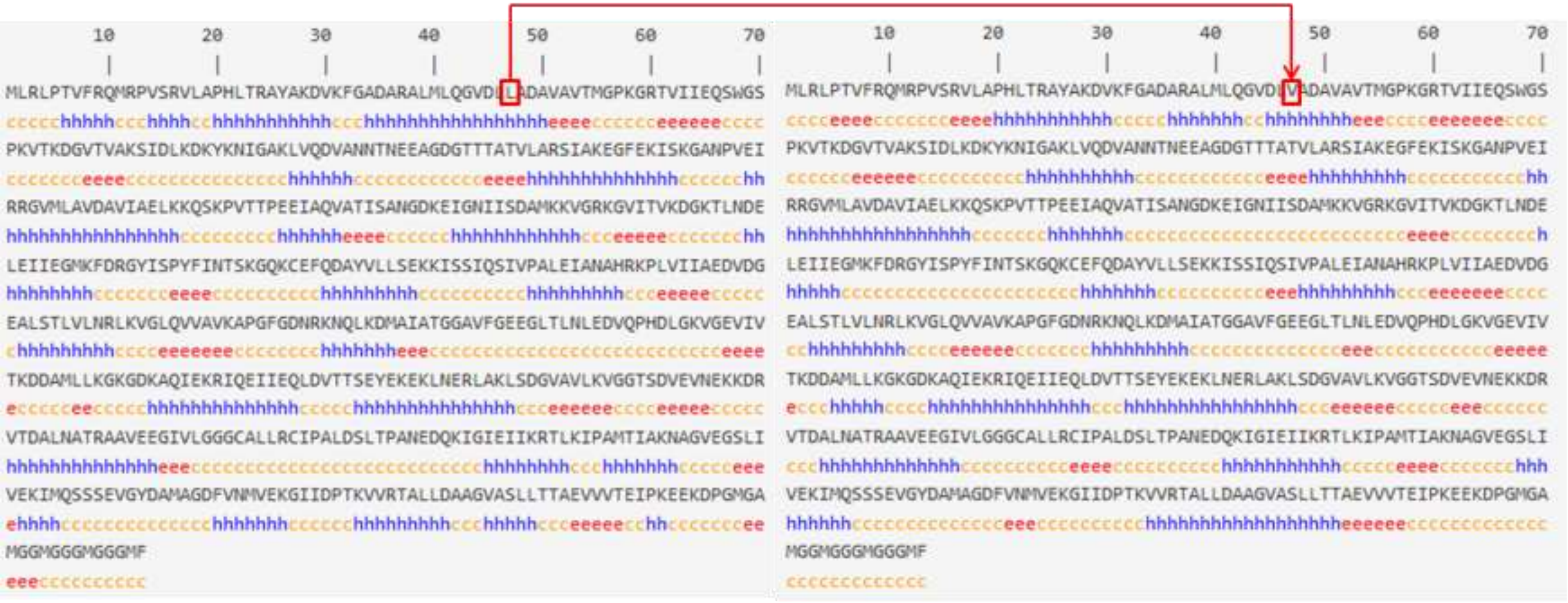

HSPD1 wild *HSPD1* p.L47V

Secondary structures are analyzed by SOPM Methods for *HSPD1* wild type and variant with p.L47V. Remarkable changes are noted between them.

Supplemental information

"Independent occurrence of *de novo* variants in *HSPD1* and *HIP1*"

Supplemental Figure S3. Results of the prediction for secondary structure of *HIP1*

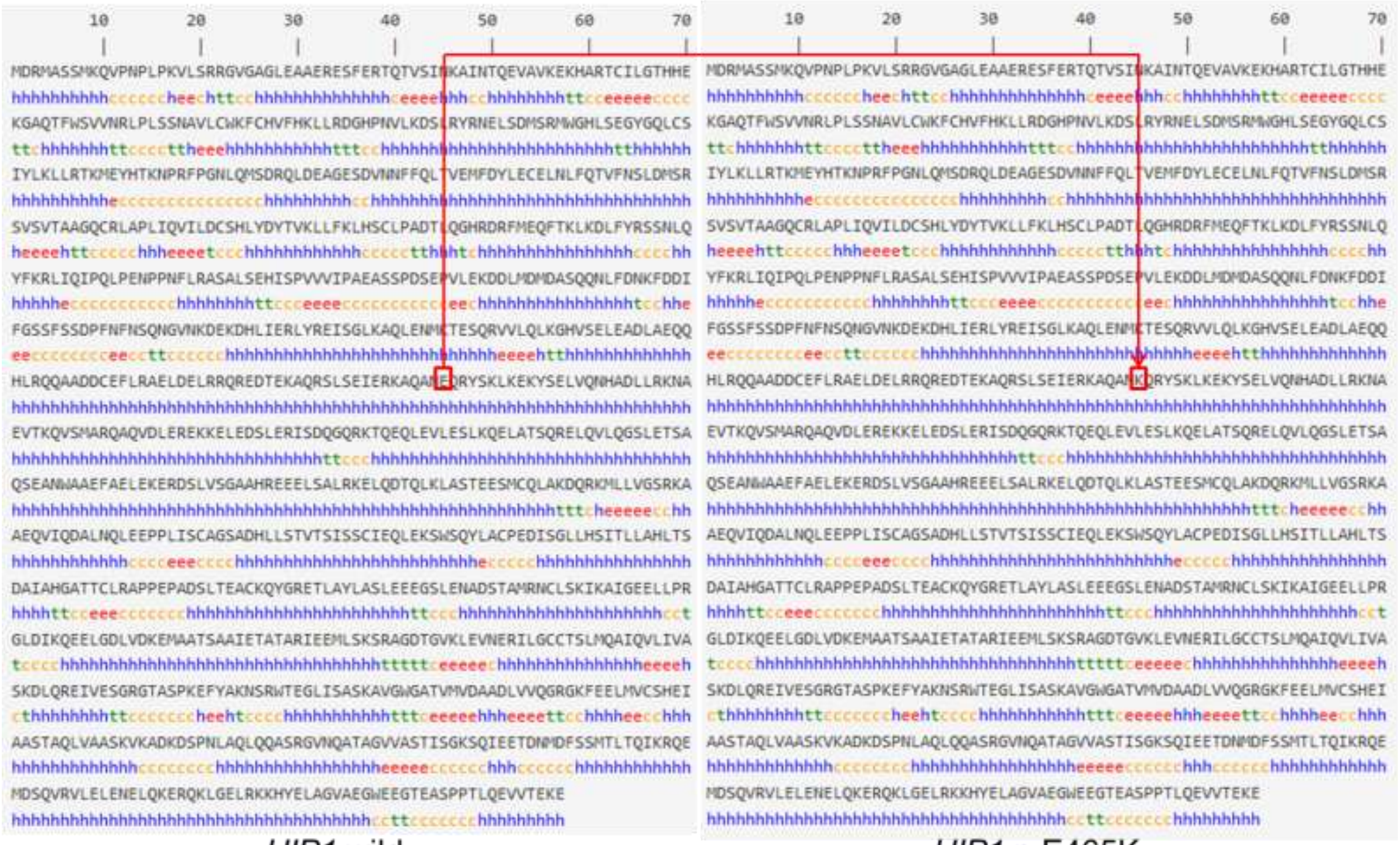

HIP1 wild

HIP1 p.E465K

No definite changes are shown between wild type and p.E465K.
